# CMR trabecular fractal analysis - technical development of a measurement system

**DOI:** 10.1186/1532-429X-16-S1-P51

**Published:** 2014-01-16

**Authors:** Gaby Captur, Vivek Muthurangu, Gherardo Finocchiaro, Filip Zemrak, Vanessa M Ferreira, Songtao Liu, Chunming Li, Steffen E Petersen, William J McKenna, Timothy J Mohun, David Bluemke, Perry M Elliott, James Moon

**Affiliations:** 1 MRI Unit, The Heart Hospital, London, UK; 2 Institute of Cardiovascular Science, University College London, London, UK; 3 UCL Centre for Cardiovascular Imaging and Great Ormond Street Hospital for Children, GOSH, London, UK; 4 Cardiac Imaging Division, The London Chest Hospital, London, UK; 5 Center for Devices and Radiological Health (CDRH)/Office of In Vitro Diagnostics and Radiological Health (OIR)/Division of Radiological Health (DIR), FDA, Silver Spring, Maryland, USA; 6 Department of Radiology, University of Pennsylvania, Philadelphia, Pennsylvania, USA; 7 Radiology and Imaging Sciences, National Institutes of Health/Clinical Center, Bethesda, Maryland, USA; 8 The Inherited Cardiovascular Disease Unit, The Heart Hospital, London, UK; 9 Department of Developmental Biology, MRC National Institutes for Medical Research, Mill Hill, UK; 10 University of Oxford Department of Cardiovascular Medicine - Centre for Clinical Magnetic Resonance Research (OCMR) The John Radcliffe Hospital Oxfordshire, The John Radcliffe Hospital, Oxfordshire, UK

## Background

Cardiac trabeculae are complex and difficult to quantify but measuring their biological signal in many cardiac diseases (left ventricular [LV] non-compaction, hypertrophic cardiomyopathy [HCM]) has value. We have recently shown how trabeculae differ between ethnicities in health and between HCM mutation carriers without hypertrophy and healthy volunteers. Here we present a novel tool for measurement for LV trabecular complexity by cardiovascular magnetic resonance (CMR) using a fractal analysis. Theoretical fundamentals are discussed. They include the measurement procedure, mathematical modeling and performance evaluation through validation by digital phantoms and reproducibility analysis when applied to real-world cardiac data.

## Methods

**Figure 1:**
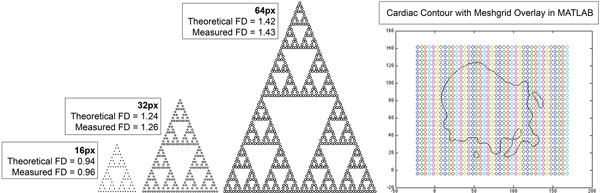
**Using the Pascal analogy triangle, we constructed mathematical fractal phantoms: Sierpinski gaskets of px heights:16-32-64-128-256 and known FD**. 16, 32 and 64 px gaskets are reproduced here(left). Predicted/measured FD demonstrate high concordance. To the right is a cardiac contour after extraction from DICOM data in MATLAB, undergoing fractal analysis. Each slice is scaled using a magnification algorithm with bicubic interpolation(Stanescu E et al.), masked and then segmented using a region-based level set method(Li C. et al). The contour, composed of N pairs of positive long X/Y coordinates, is overlaid by a series of meshgrids, replicated over 4 random start points. Starting box-size = 45% that of the minimum circumscribed box with parallel constraint; terminal box-size = 4 px to maintain stable regression. Box size decreases by one px per iteration. At every level, scale and box count are recorded and formulated into a natural logarithmic plot. The slope determines the FD. Px = pixel.

The measurement system consists of an in-house developed software written in MALTAB R2012b that parametrizes 2-dimensional LV cine end-diastolic short-axis frames in DICOM image format. Images are scaled, masked and segmented before undergoing fractal analysis. Endocardial contours serve as the basis for estimation of the fractal dimension (FD) -a unitless index of endocardial complexity, calculated using a box-counting algorithm which cycles through a series of grids of boxes overlying the region of interest. Validation was by digital phantoms consisting of planar targets of known FD (Figure 1) generated in MATLAB. The procedure was further evaluated in terms of intra- and inter-observer variability of calculated maximal apical FD in 45 randomly-selected scans from within a research CMR database (all 1.5-T, Siemens and General Electric) and in terms of inter-study reproducibility (20 repeat scans, same 1.5T magnet; mixed healthy/diseased cohort). Impact of varying slice thickness (43 cine slices repeated at thicknesses: 8, 7, 6 mm) and magnetic field strength (134 cine slices repeated at 1.5 and 3T; mixed healthy/diseased cohort) on raw slice-by-slice FD was also evaluated.

## Results

**Figure 2:**
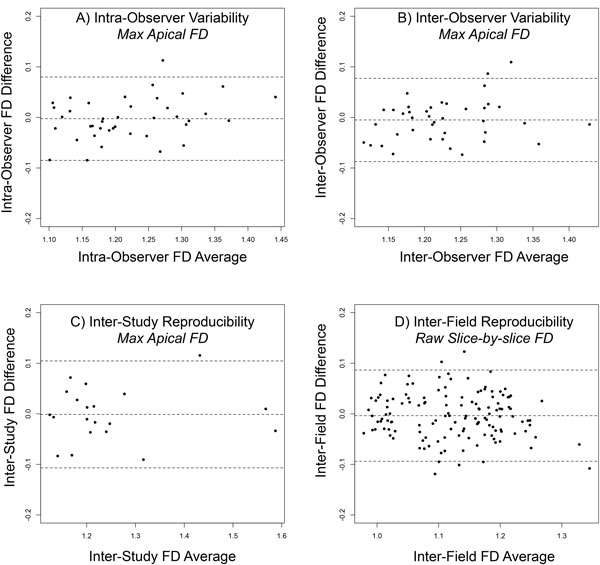
**Scatter plots showing difference in FD measurements against average of the repeated values for: A) intra-observer variability; B) inter-observer variability; C) inter-study reproducibility; D) inter-field reproducibility**. There is no systematic bias. Middle line = mean difference, two extreme lines = limits of agreement (+1.96 and -1.96 standard deviation).

Phantom validation of the box-counting algorithm produced measured FD which closely approximated expected values as proof of reliability. Intra- and inter-observer variability for maximal apical FD was high (ICCs: 0.94, 95%CI 0.90-0.97 and 0.92, 95%CI 0.86-0.95 respectively; Figure 2A, B) as was inter-study reproducibility (ICC 0.96, 95%CI 0.83-0.98; Figure 2C). ANOVA with repeated measures determined that mean FD did not differ significantly between the 3 slice thicknesses (F(2, 84) = 2.259, p = 0.1). Agreement between raw FD values was good irrespective of magnetic field strength (ICC 0.92, 95%CI 0.90 0.94; Figure 2D).

## Conclusions

This is the first comprehensive implementation of a fractal procedure applied to the clinical CMR domain. Fractal analysis provides a way of collapsing the morphometric complexity of LV trabeculae into a single numerical value which can be compared between groups of subjects. Here we show how fractal analysis is a robust, reliable and reproducible method for capturing the richness of cardiac trabeculae imaged by CMR.

## Funding

Dr Captur is funded by the University College London (Graduate Research Scholarship) and by the European Union (Science and Technology Research Grant). Dr Moon is funded by the Higher Education Funding Council for England.

